# Significance of Oncocytic Cells in Thyroid Fine Needle Aspiration Specimens Classified as Atypia of Undetermined Significance

**DOI:** 10.5146/tjpath.2026.14128

**Published:** 2026-01-31

**Authors:** Aysegul Aksoy Altınboga, Elif Dogan Kabadayı, Tuba Dilay Kokenek Unal, Nur Gizem Kocaoglu Celik, Serhat Ozan, Asiye Safak Bulut, Cevdet Aydın

**Affiliations:** Department of Pathology, Ankara Yildirim Beyazit University, Faculty of Medicine, Ankara, Türkiye; Ankara Bilkent City Hospital, Ankara, Türkiye; Ankara Bilkent City Hospital, Ankara, Türkiye; Department of Endocrinology and Metabolism, Ankara Yildirim Beyazit University, Faculty of Medicine, Ankara, Türkiye

**Keywords:** Thyroid, Fine needle aspiration, Oncocytes, Hürthle cells, Atypia of undetermined significance

## Abstract

**Objective:** Oncocytic cells are commonly detected on FNA reports but little is known regarding the relationship between the degree of oncocytic cell features and the rate of malignancy (ROM). In this study, we aimed to investigate the importance of oncocytic cells in thyroid FNA with a diagnosis of atypia of undetermined significance (AUS). Additionally, we sought to ascertain if the prevalence of oncocytic cells in FNAs with oncocytic cells is linked to neoplasm and malignancy.

**Material and Methods:** 187 cases belonging to 144 patients diagnosed with AUS in thyroid FNA were re-evaluated for the oncocytic cells, nuclear atypia and microfollicles and then classified as AUS-nuclear with or without oncocytic cells and AUS-other with or without oncocytic cells. The cases that had oncocytic cells were scored according to the proportion of oncocytic cells.

**Results:** ROM was higher in the AUS-nuclear group (28.1%) compared to the AUS-other group (12.2%). AUS-nuclear cases without oncocytic cells had higher ROM compared to the AUS-nuclear cases with oncocytic cells. Rate of neoplasm (RON) was significantly higher in the cases containing 75% or more oncocytic cells than the other cases (p<0.0001). (26.3% for cases with 1–75% oncocytic cells, 81.3% for those with 75% or more oncocytic cells).

**Conclusion:** This study showed that the AUS cases with a dominant oncocytic cell population might be more specific for neoplastic processes. Presence of oncocytic cells in the AUS-nuclear cases causes a decrease in ROM. Emphasizing oncocytic cells in the report may contribute to patient follow-up and treatment in AUS.

## INTRODUCTION

Thyroid nodules are now a widely recognized clinical problem due to the widespread use of imaging techniques (1). The clinical significance of thyroid nodules is based on the need to rule out thyroid cancer, which occurs in 5–15% of cases (1). The Bethesda System Reporting Thyroid Cytology (BSRTC), a standard diagnostic language in thyroid FNAs, has evolved over time to aid in the differential diagnosis of thyroid nodules (2,3). The BSRTC has six diagnostic categories, each with specific criteria and the associated risk of malignancy (1). Diagnostic categories in BSRTC include non-diagnostic (ND), benign (B), atypia of undetermined significance (AUS), follicular neoplasm (FN), suspicious for malignancy (SFM) and malignant (M).

Hürthle cells/oncocytic cells are large cells with large eosinophilic granular cytoplasm that develop from thyroid follicular epithelial cells (4). Oncocytic cells are present in a broad range of thyroid gland lesions, from benign and malignant neoplasms like oncocytic adenoma and oncocytic carcinoma to numerous non-neoplastic benign conditions including lymphocytic thyroiditis, follicular nodular disease, and radiation therapy to the head and neck area (5–8). In the WHO 2023 classification, the term “oncocytic carcinoma of the thyroid” is used to describe follicular cell-derived tumors that contain at least 75% oncocytic cells, lack high-grade characteristics, and lack PTC nuclear features (9).

Oncocytic PTC, oncocytic encapsulated follicular subtype of PTC, oncocytic poorly differentiated carcinoma, and oncocytic medullary thyroid carcinoma are among the various subtypes of oncocytic follicular cell-derived thyroid carcinomas other than “oncocytic carcinoma of the thyroid” (10,11).

Oncocytic cells are commonly detected on FNA reports, but physicians may be unsure of how to interpret the presence of oncocytic cells in relation to the BSRTC categorization and view the presence of oncocytic cells indicating “atypical cells” (12). Many clinicians/medical professionals believe that the existence of oncocytic cells raises the risk of cancer above what a certain BSRTC category would have suggested (12). Furthermore, there can be wide variations in the degree of oncocytic cells in FNA cytology. However, little is known regarding the relationship between the degree of oncocytic cell features and the rate of malignancy on final pathology (7).

In this study, we aimed to investigate the importance of oncocytic cells in thyroid FNA with a diagnosis of atypia of undetermined significance and histopathologic follow-up. Additionally, we sought to ascertain if the prevalence of oncocytic cells in FNAs with oncocytic cells is linked to neoplasm and malignancy.

## MATERIALS AND METHODS

The study was approved by the institutional Ethics Committee on March 2025 with the TABED1-25-1135 numbered board decision. In this study, the thyroid fine needle aspiration cytologies between February 2019 and December 2020 were scanned from the hospital database and 203 cases of AUS with histopathologic correlation were identified. Cases that did not have cytological or histopathological slides in the archive, were categorized in a diagnosis other than AUS on re-evaluation, and had less than 100 cells in smears were excluded from the study. After sixteen patients were excluded, 187 cases belonging to 144 patients diagnosed with AUS on thyroid FNA were identified.

### Cytologic Evaluation

MGG-stained FNA smears were re-evaluated and re-classified according to nuclear and architectural atypia criteria by 2 cytopathologists (AAA and SC) according to BSRTC. The presence of oncocytic cells was investigated in each case. Cases were divided into 4 groups according to the presence of oncocytic cells, nuclear atypia, and microfollicles;

1. AUS-N: Cases with nuclear atypia and no oncocytic cells

2. AUS-N-O: Cases containing oncocytic cells with nuclear atypia

3. AUS-A: Cases with microfollicles without nuclear atypia and without oncocytic cells

4. AUS-O: Cases without nuclear atypia, containing oncocytic cells, with or without microfollicles

**Figure 1:**
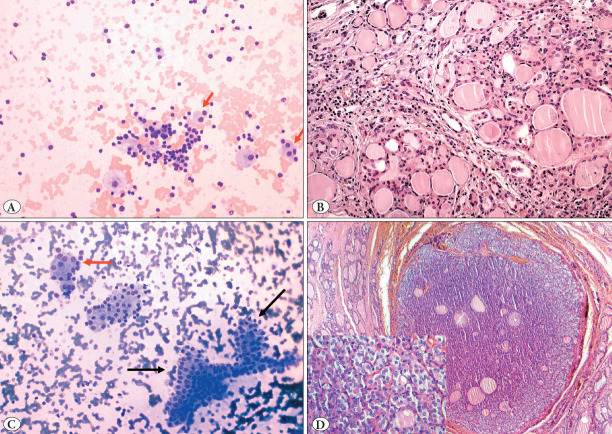
AUS cases with score 1 (1-25%) oncocytic cells: A) Smear with low cellularity showing benign thyrocyte clusters, few oncocytes (red arrow) and a few macrophages, (MGGx200) **B)** Oncocytic nodular hyperplasia on the background of chronic lymphocytic thyroiditis on thyroidectomy section of the smear seen in picture A, (H&Ex200) **C)** A small cluster of oncocytic cells (red arrow), a cluster of benign thyrocytes and an atypical cluster of thyrocytes with slightly elongated nuclei and nuclear overlap (black arrow), (MGGx200) **D)** Noninvasive follicular tumor with papillary like nuclear features (NIFTP) in thyroidectomy section of the smear seen in picture C (H&Ex40, inset H&Ex400).

Cases were then scored according to the proportion of oncocytic cells:

Score 1: those containing 1-25% oncocytic cells (Figure 1)

**Figure 2:**
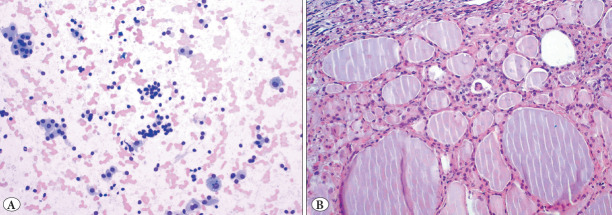
A case with score 2 (26-74%) oncocytic cells **A)** Oncocytic cell groups mixed with benign follicular epithelial cell clusters (MGGx200), **B)** Nodular hyperplasia showing oncocytic metaplasia on thyroidectomy section.

Score 2: containing 26-74% oncocytic cells (Figure 2)

**Figure 3:**
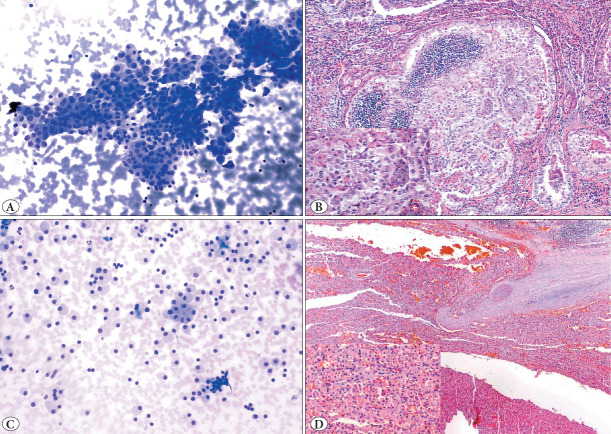
Cases with score 3 (>75%) oncocytic cells **A)** A large cluster of atypical oncocytic cells with large and elongated nuclei and nuclear overlap (MGGx200), **B)** Oncocytic papillary thyroid carcinoma in the thyroidectomy section of picture A (H&Ex40, inset: H&Ex400), **C)** The smear is cellular and almost entirely composed of oncocytic cells, with few lymphocytes in the background.Clinical, radiologic and laboratory findings of the patient were consistent with lymphocytic thyroiditis. (MGGx200) **D)** Thyroidectomy section of Picture C shows oncocytic carcinoma with capsular invasion. Recurrence occurred 1 year later. (H&Ex40, inset: H&Ex100).

Score 3: containing *≥*75% oncocytic cells (Figure 3)

Since the presence of 75% or more oncocytic cells in BRSTC is considered significant in terms of a neoplastic process, scores 1 and 2 were grouped together and cases were statistically analyzed according to whether they contained 75% or less oncocytic cells. Score 3 cases included sparsely cellular aspirates formed primarily or nearly completely of oncocytic cells with minimal colloid, or the specimens which were composed exclusively of oncocytic cells in a patient with Hashimoto thyroiditis or multinodular goiter (MNG).

Rate of neoplasia (RON) and rate of malignancy (ROM) were calculated separately for each diagnostic group and for the groups divided according to oncocytic cell rates.

The number of cases with surgical follow-up divided by the total number of histopathologically proven malignant cases yielded the ROM. Noninvasive follicular thyroid neoplasm with papillary-like nuclear features (NIFTP) is not regarded as being identical to carcinoma in the context of ROM calculations. Histologically verified benign neoplasms were also included in the numerator for the RON.

The sections of the thyroidectomies were re-evaluated (AAA and NGKÇ) and classified as benign, low-risk neoplasms and malignant neoplasms according to the WHO 2023 classification (9).

### Statistical Analysis

The statistical analysis was performed using SPSS 22.0 for Windows (SPSS, Inc.; Chicago, IL. USA). Descriptive statistics were defined as number (n), percentage (%), mean ± standard deviation, or median with IQR values. The normal distribution of data was evaluated using histograms, q-q plots, and the Shapiro-Wilk’s test. The categorical variables were analyzed using the Pearson chi-square and Fisher tests. Statistics were deemed significant if the p value was <0.05 at the 95% confidence interval.

## RESULTS

The study includes 187 FNAs of 144 patients (45 (30.8%) males and 99 (69.1%) females) with a mean age of 49.3 years (range: 15-76 years). Cytologic evaluation revealed 98 (52.4%) AUS-N, 48 (25.7%) AUS-N-O, 25 (13.4%) AUS-O, and 16 (8.5%) AUS-A cases.

The distribution of histopathologic diagnosis in thyroidectomies is as follows: 90 (62.5%) benign, 36 (25%) malignant neoplasm, and 18 (12.5%) low-risk neoplasm. The distribution of cytopathologic and histopathologic diagnoses are shown in Table 1.

**Table 1:** Distribution of cytopathologic and histopathologic diagnoses of the cases

**Histopathologic diagnosis**		**Cytopathologic diagnosis**				**Total** **n**
		**AUS-Nuclear**		**AUS-Other**		
		**AUS-N** **n (%)**	**AUS-N-O** **n (%)**	**AUS-O** ** n (%)**	**AUS-A** **n (%)**	
Benign	FND	17 (17.4)	5 (10.4)	3 (12)	1 (6.3)	26
	FND+ LT	29 (29.6)	19 (39.6)	6 (24)	5 (31.3	59
	LT	2 (2)	9 (18.7)	3 (12)	1 (6.2)	15
	FA	4 (4.1)	5 (10.4)	8 (32)	4 (25)	21
Malignant	PTC	35 (35.7)	5 (10.4)	3 (12)	2 (12.5)	45
	OC	-	1 (2.1)	-	-	1
Low risk neoplasms	NIFTP	7 (7.2)	2 (4.2)	2 (8)	3 (18.7)	14
	WDT-UMP	2 (2)	1 (2.1)	-	-	3
	FT-UMP	2 (2)	1 (2.1)	-	-	3
Total		98	48	25	16	187

**FND:** follicular nodular disease, **LT:** lymphocytic thyroiditis, **FA: **follicular adenoma, **PTC:** papillary thyroid carcinoma, **OC: **oncocytic carcinoma, **NIFTP:** noninvasive follicular tumor with papillary-like nuclear features, **WDT-UMP: **well-differentiated tumor of uncertain malignant potential, **FT-UMP:** follicular tumor of uncertain malignant potential, **AUS-N: **Atypia of undetermined significance-Nuclear, **AUS-N-O:** Atypia of undetermined significance-Nuclear-with oncocytic cells, **AUS-0: **Atypia of undetermined significance-with oncocytic cells, without nuclear atypia, **AUS-A:** Atypia of undetermined significance-with architectural atypia, without nuclear atypia and oncocytic cells

In the histopathologic follow-up of the AUS-N group, 52 benign cases (53.1%), 35 malignant neoplasms (35.7%), and 11 low risk neoplasms (11.2%) were found. In the histopathologic follow-up of the AUS-N-O group, 38 benign cases (79.1%), 6 malignant neoplasms (12.5%), and 4 low risk neoplasms (8.4%) were identified. In the histopathologic follow-up of the AUS-O group, there were 20 benign cases (80%), 3 malignant neoplasms (12%), and 2 low risk neoplasms (8%). In the AUS-A group, 11 benign cases (68.8%), 2 malignant neoplasms (12.5%), and 3 low risk neoplasms (18.7%) were found. The detailed cytopathologic and histopathologic diagnostic distribution of the cases is shown in Table 1.

RON and ROM were calculated for each diagnostic group.

RON and ROM were found to be higher in the AUS-N group compared to the AUS-N-O group and this difference was statistically significant (p=0.033 for RON and p=0.003 for ROM). No statistically significant difference was found between AUS-O and AUS-A, AUS-N-O and AUS-O, and AUS-N and AUS-A in terms of RON and ROM (p>0.05) (Table 2).

**Table 2:** Rate of neoplasia and rate of malignancy in each diagnostic group and overall

	**Cytopathologic diagnosis**				**Total**	**p**
	**AUS-Nuclear**		**AUS-Other**			
	**AUS-N**	**AUS-N-O**	**AUS-O**	**AUS-A**		
RON n (%)	50 (51)	15 (31.3)	13 (52)	9 (56.3)		*p=0.033, **,***,****p>0.05
ROM n (%)	35 (35.7)	6 (12.5)	3 (12)	2 (12.5)		*p=0.003 **,*** ,****p>0.05
RON n (%)	65/146 (44.5)		22/41 (53.7)		87 (46.5)	0.376
ROM n (%)	41/146 (28.1)		5/41 (12.2)		46 (24.6)	0.041
Total	98	48	25	16	187	

**RON:** rate of neoplasm, **ROM: **rate of malignancy, **AUS-N:** Atypia of undetermined significance-Nuclear, **AUS-N-O:** Atypia of undetermined significance-Nuclear-with oncocytic cells, **AUS-0:** Atypia of undetermined significance-with oncocytic cells, without nuclear atypia, **AUS-A: **Atypia of undetermined significance-with architectural atypia, without nuclear atypia and oncocytic cells, *: p of AUS-N and AUS-N-O, **: p of AUS-O and AUS-A, *** p of AUS-N and AUS-A, ****: AUS-N-O and AUS-O

When the AUS-nuclear (AUS-N + AUS-N-O) and AUS-other (AUS-O + AUS-A) groups were evaluated in terms of RON and ROM, ROM was found to be higher in the AUS-nuclear group compared to the AUS-other group and this difference was statistically significant (p=0.041). There was no statistically significant difference between AUS-nuclear and AUS-other in terms of RON (p>0.05), (Table 2).

Considering all groups, RON and ROM were found to be 46.5% and 24.6%, respectively, in all AUS cases included in the study (Table 2).

Oncocytic cell ratios and histopathologic follow-up information of the cases with oncocytic cells are shown in Table 3. Scores 1 and 2 were evaluated as a single group during statistical analysis, and cases were divided into 2 groups as cases containing less than 75% oncocytic cells and 75% or more oncocytic cells.

**Table 3:** Histopathologic follow-up of the cases according to percentages of oncocytic cells

**Oncocytic cells**		**Cytopathologic diagnosis**			
		**AUS-Nuclear**		**AUS-Other**	
		**AUS-N-O** **[n (%)]**	**Histopatologic follow-up**	**AUS-O** **[n (%)]**	**Histopatologic follow-up**
Numbers (%)	1-25	11 (22.9)	5 benign, 2 low-grade neoplasm, 4 malignant	12 (48)	10 benign, 2 malignant
	26-75	28 (58.3)	26 benign, 1 low-grade neoplasm, 1 malignant	6 (24)	5 benign, 1 malignant
	>75	9 (18.8)	7 benign, 1 low-grade neoplasm, 1 malignant	7 (28)	5 benign, 2 low grade neoplasm
Total		48 (100)		25 (100)	

**FND:** follicular nodular disease, **LT:** lymphocytic thyroiditis, **FA: **follicular adenoma, **PTC:** papillary thyroid carcinoma, **FVPTC:** follicular variant of papillary thyroid carcinoma, **OC:** oncocytic carcinoma, **NIFTP: **noninvasive follicular tumor with papillary-like nuclear features, **WDT-UMP: **well-differentiated tumor of uncertain malignant potential, **FT-UMP: **follicular tumor of uncertain malignant potential, **AUS:** Atypia of undetermined significance, **AUS-N-O: **Atypia of undetermined significance-Nuclear-with oncocytic cells, **AUS-0:** Atypia of undetermined significance-with oncocytic cells, without nuclear atypia

RON was higher in the AUS-N-O group containing more than 75% oncocytic cells compared to the AUS-N-O group containing less than 75% oncocytic cells and this difference was statistically significant (p=0.018). Whether the oncocytic cell ratio was below or above 75% did not cause a statistically significant difference in ROM values in the AUS-N-O group (p>0.05) (Table 4).

**Table 4:** Rate of neoplasia and rate of malignancy according to presence and percentages of oncocytic cells

**Oncocytic cells**		**Cytopathologic diagnosis**						**Total** **N (%)**
		**AUS-Nuclear**			**AUS-Other**			
		**AUS-N-O [n (%)]**			**AUS-O [n (%)]**			
Numbers (%)	1-75	39 (81.2)	RON	9 (23.1)	18 (72)	RON	6 (33.3)	15/57 (26.3)
			ROM	5 (12.8)		ROM	3 (16.7)	8/57 (14.0)
	>75	9 (18.8)	RON	6 (66.7)	7 (28)	RON	7 (100)	13/16 (81.3)
			ROM	1 (11.1)		ROM	0	1/16 (6.3)
Total		48 (100)	RON	15 (31.2) p=0.018	25 (100)	RON	13 (52) p=0.005	28/73 (38.4) p < 0.0001
			ROM	6 (12.5) p>0.05		ROM	3 (12) p>0.05	9/73 (12.3) p>0.05

**AUS:** Atypia of undetermined significance, **AUS-N-O: **Atypia of undetermined significance-Nuclear-with oncocytic cells, **AUS-0:** Atypia of undetermined significance-with oncocytic cells, without nuclear atypia, **RON:** rate of neoplasia, **ROM:** rate of malignancy

RON was higher in the AUS-O group containing more than 75% oncocytic cells, than the AUS-O group containing less than 75% oncocytic cells. This difference was statistically significant (p=0.005). The increase in the proportion of oncocytic cells in the AUS-O group did not cause a statistically significant difference in ROM values (p>0.05) (Table 4).

All cases containing oncocytic cells (AUS-N-O + AUS-0) were further evaluated in terms of oncocytic cell rates (below and above 75%). RON was significantly higher in the cases containing 75% or more oncocytic cells than the cases containing less than 75% oncocytic cells, and this difference was statistically significant (p<0.0001). There was no statistically difference in the ROM values according to the proportion of oncocytic cells (p>0.05) (Table 4).

In all AUS cases with oncocytic cells, RON was 38.4% (28/73) and ROM was 12.3% (9/73) when evaluated independently of the percentage of oncocytic cells and based only on their presence (AUS-N-O and AUS-O).

## DISCUSSION

According to the results of this study, ROM values change according to the presence or absence of oncocytic cells in the AUS-nuclear group, and the presence of oncocytic cells in the AUS-nuclear group decreases ROM significantly. There is also a statistically significant difference in RON values between these two groups. When the percentage of oncocytic cells was 75% and above, a statistically significant increase in RON was observed in both AUS-nuclear, AUS-other, and all AUS cases overall. This difference is more pronounced in the AUS-O group without nuclear atypia.

Specimens that have one or more of a diverse set of findings that raise concerns about neoplasm or malignancy but are not enough to be categorized as a follicular neoplasm, suspicious for malignancy, or malignant fall under the diagnostic category known as “Atypia of Undetermined Significance” (AUS). The most common causes of AUS are either a predominance of oncocytic cells or atypia in follicular cells, which are usually nuclear and/or architectural in nature (13).

Since the BSRTC was first defined in 2007, there have been numerous studies on the AUS category. Although the risk of malignancy in the AUS category is highly variable (10-37%), a decrease has been observed since the definition of NIFTP (2,14,15). In the present study, which evaluated only AUS cases with histopathologic follow-up, ROM was 24.6% when all AUS groups were evaluated together before subdividing into subgroups.

Compared to studies assessing ROM, there is less research in the literature on the RON in AUS patients as determined by BSRTC. According to Guleria et al., RON was 68.6% in AUS cases (14). When all AUS cases were taken into account, we discovered that RON was 46.5% in our study.

ROM rates in the AUS category were also examined in the literature based on subgroups. ROM varies according to the AUS/FLUS subclassification in numerous studies (16,17). The AUS subcategory of cytologic/nuclear atypia frequently displayed a higher ROM than other AUS subcategories, ranging from 34.4% to 97%. This is in contrast to the ROM range for the subcategories of architectural atypia (5%-26.9%) and predominantly Hürthle cells (5%-22.2%) (12,15–26). In the light of these data, it was recommended to report cases in the AUS group as AUS-nuclear and AUS-other in the BSRTC 3rd edition (13).

In our study, we evaluated AUS cases primarily in two groups as AUS-nuclear and AUS-other in accordance with the BSRTC 3rd edition. We found ROM as 28.1% for AUS-nuclear and 12.2% for AUS-other. Consistent with the literature, the ROM value was found to be higher in the AUS-nuclear group than in the AUS-other group in our study with a statistically significant difference (15).

The impact of oncocytic cell presence on malignancy rates within the BSRTC diagnostic categories is not well discussed in the literature (12). Oncocytic follicular neoplasm is defined as a moderately or significantly cellular aspirate from a single nodule that is almost entirely made up of oncocytic cells (13). In cases where a sparsely cellular aspirate is formed primarily or nearly completely of oncocytic cells with minimal colloid, or when a specimen is composed exclusively of oncocytic cells in a patient with Hashimoto thyroiditis or MNG, AUS is the preferred diagnostic category. Smears containing oncoytic cells are recommended to be reported as AUS-other in the third edition of the BSRTC (13).

The primary goal of this study was to find out how oncocytic cell presence affected the rate of malignancy and rate of neoplasm in AUS cases. Because of this, the AUS-nuclear and AUS-other groups were then assessed independently based on whether or not they contained oncocytic cells, and the proportions of oncocytic cells were also disclosed in the current study. The overall malignancy risk for the AUS/FLUS category was 15% in a study that assessed the risks of malignancy in all BSRTC categories with Hürthle cells and with histopathologic correlation (12). According to their research, the Hürthle cell presence overall did not increase the risk of malignancy. On the other hand, ROM rose to 21% without a statistically significant change when the Hürthle cells predominated (12). ROM was reported to be 32% in AUS cases in another study examining the effect of Hürthle cells on thyroid FNAs in which Hürthle cells were predominant (26). The range of ROM values in AUS with Hürthle cells has been found to be between 9.7% and 29% in several studies (4,15,16,27–29). ROM was 12.3% in our study when AUS cases with oncocytic cells (AUS-N-O and AUS-O) were assessed together, and this rate is lower than the majority of studies.

In the literature, RON for AUS cases with oncocytic cells ranges from 37% to 77.8% (16,26,27). In our study, when AUS patients containing oncocytic cells (AUS-N-O and AUS-O) were assessed together, regardless of the proportion of oncocytic cells, a lower RON (38.4%) was found regardless of the proportion of oncocytic cells.

The AUS cases in our study were separated into two categories: nuclear and other. They were then assessed independently based on whether or not they included oncocytic cells. When oncocytic cells were present in the smears of AUS-nuclear cases, a decrease in ROM was observed. This implies that the inclusion of oncocytic cells in the report may contribute to patient follow-up and treatment in AUS-nuclear cases. In some of these individuals, the presence of oncoytic cells in the AUS-nuclear group gives the clinician the opportunity to prevent an unnecessary lobectomy. Clinical, sonographic, and molecular correlation will frequently be the basis for the clinical decision in this situation to monitor a patient instead of doing a lobectomy.

We also investigated the effect of the percentage of oncocytic cells (<75% or *≥*75%) on the detection of malignancy and neoplasia. Oncocytic cells ‘at or above 75%’ were shown to raise RON in both the AUS-nuclear (AUS-N-O) and AUS-other (AUS-O) categories. For all AUS patients with oncocytic cells, the RON was 38.4%; for those with 1–75% oncocytic cells, it was 26.3%; and for those with 75% or more oncocytic cells, it was 81.3%. The difference between the groups is statistically significant. However, the percentage of oncocytic cells did not cause any change in ROM values.

Our study has a number of limitations. Our report introduces institutional bias, including referral bias, surgeon prejudice, and assessment bias, because it is based on a single cohort from a single institution. Second, only those who had the associated thyroid nodule surgically removed later are included in the study.

In conclusion, the BSRTC proposes to report the AUS group as AUS-nuclear and AUS-other. However, the AUS cases with a dominant oncocytic cell population might be more specific for neoplastic processes. In this instance, doing a lobectomy should be a more acceptable course of action than monitoring a patient who exhibits clinical, sonographic, and/or relevant molecular changes. Oncocytic cell prominence should therefore be reported separately. The role of oncocytic cells in AUS should be further understood in future research that includes AUS patients with clinical or surgical follow-up and molecular studies.

## Conflict of Interest

No conflict of interest was declared by the authors.

## Funding

The authors declared that this study received no financial support.

## Ethical Approval

The study was approved by the Ethics Committee of Ankara Bilkent City Hospital with the board number TABED1-25-1135 dated March, 2025.
